# From lab to lifesaver: the rise of CAR T-cell therapy in oncology

**DOI:** 10.1186/s43046-025-00262-6

**Published:** 2025-05-16

**Authors:** Shriyash S. Jangavali, Pallavi B. Hangargekar, Balasaheb U. Gangthade, Shreya A. Jadhav, Ujwal A. Havelikar, Amol A. Joshi

**Affiliations:** 1 Department of Pharmacy, ASPM’s K. T. Patil College of Pharmacy, Dharashiv, 413501 Maharashtra India; 2 Department of Quality Assurance, ASPM’s K. T. Patil College of Pharmacy, Dharashiv, 413501 Maharashtra India; 3 Department of Pharmacy, NIMS Institute of Pharmacy, Jaipur, Rajasthan India; 4 Department of Pharmacognosy, ASPM’s K. T. Patil College of Pharmacy, Dharashiv, 413501 Maharashtra India

**Keywords:** Chimeric antigen receptor, Oncology, Cancer immunotherapy, Cytotoxic release syndrome, CAR T-cell therapy

## Abstract

**Background:**

Recently, cancer treatment paradigms have shifted dramatically with the advent of immunotherapies, particularly chimeric antigen receptor (CAR) T-cell therapy. Despite it is revolutionary positive outcomes in treating hematologic malignancies, challenges such as severe toxicities, high treatment costs, and limited efficacy in solid tumors persist. This review highlights these limitations and the ongoing need for innovation in CAR T-cell therapy.

**Main body:**

This manuscript provides a comprehensive review of most current advancements in CAR T-cell therapy, with a focus on targeting its immunotherapeutic principles, modification of T cells for Targeted cancer therapy using T cells, and clinical applications. It explores the key elements of CAR T-cell therapy, containing antigen recognition domain and intracellular signaling domains, which enable T cells to interact with cancer cells and exert cytotoxic effects. The review examines approved therapies, and ongoing clinical trials, Along with obstacles like cytokine release syndrome (CRS), neurotoxicity, along antigen escape mechanisms. Furthermore, innovations in cutting-edge CAR T-cell therapies and personalized treatment approaches are discussed, together with an emphasis on improving safety and efficacy.

**Conclusion:**

The manuscript outlines the future outlook on integrating CAR T-cell therapy integrated with other treatments and exploring patient-specific approaches to revolutionize cancer care. This review aims to bridge the existing gaps in research, offering valuable insights for students and researchers in biomedical sciences and oncology.

## Introduction

With rising yearly incidence and fatality rates, cancer poses a serious health risk [1]. The cornerstone of cancer treatment for many years has involved the combination of newly evolved targeted medicines As well as conventional therapies as in the case of radiation, surgical intervention, and chemotherapy. Although they have shown promise, several cancers have poor prognoses that have been linked to treatment for associated consequences [2]. The therapeutic view of human cancers has evolved within the past ten years with the introduction of various innovative therapeutic strategies. The application of immunotherapy has revolutionized the field of cancer treatments [3]. One highly successful cellular immunotherapy approach for cancer treatment involves the engineering of immune cells for the production of cell surface receptors that might recognize and remove antigens found on the surface of tumor cells [1]. Successfully treating hematological cancers, CAR-T cell therapy is identified as a leading immunotherapy technique [3].

The effects of CAR-T cell therapy are exceptional [4]. The fusion proteins produced by CAR-T cells direct T cells to a particular antigen found in tumor cells so that the immune system can mount an anticancer defense [5]. Composed of four fused domains, the CAR protein is modular. The components comprise the extracellular target-binding domain, spacer domain, transmembrane domain, and CD3z6, generally sourced from different segments of a single-chain antibody [scFv] [6].

CAR-T cell therapy offers a breakthrough in the management of cancer as the FDA has currently approved axicabtageneciloleucel and other CD19-targeted CAR-T cell medicines. LisocabtageneMaraleucel, Tisagenlecleucel, and Brexucabtagene Autoleucel. The FDA has furthermore given its approval to idecabtagenevicleucel and ciltacabtageneautoleucel, two BCMA-targeted CAR-T cell products. All of these products are made by virally expressing the CAR design on autologous [patient-derived] T cells [5, 7]. CAR-T adoptive immunotherapy has yielded several clinical benefits for the treatment of human cancers. By using this technique, T cells are modified to recognize membrane antigens specific to tumors and subsequently eliminate cancerous cells [3]. To optimize the potential benefits of over-the-counter therapies, as seen in gene-edited CAR-T, it will be crucial to establish production strategies that might increase the production of CAR-NK products to treat the greatest number of patients in each batch [8].

In general, cancer immunotherapy aims to eradicate cancer cells by stimulating the immune system. One type of cellular immunotherapy is called CAR-T cell treatment. To target tumor antigens, The CAR-T treatment process includes the removal of T cells derived from a patient’s blood, enhancing them for expressing artificial antigen receptors, and then mixing these cells [9].

Patients with tumors that are resistant to conventional therapy techniques may be cured with the help of genetically modified CAR-T cells. Adoptive cell treatments have not been as successful against solid cancers thus far [10]. The cornerstone of antiviral and anticancer processes is the immune system [11]. Viral delivery techniques have been necessary to genetically Modify T cells or other immune cells for CAR-T generation cells [12].

For the purpose of use with CAR-T cell therapy, CAR-T cells undergo genetic modification to identify and target specific cancer cells. Due to the shortcomings of traditional oncological treatments including radiation and chemotherapy, which frequently have serious side effects and might not completely eradicate all cancer cells, such novel medications are needed. With the potential to completely change how cancer is treated, CAR-T cell therapy offers a personalized and efficient replacement [13]. The hunt for more accurate and minimally invasive cancer treatments has been sparked by the effectiveness of traditional cancer treatments such as radiation and chemotherapy, despite their limits in focusing on particular cancer cells and possible side effects. Depending on the idea of utilizing the immune system's inherent powers, immunotherapy has become well-known as a potentially effective treatment option. Immunotherapy targets the body’s immune response to detect and destroy cancer cells by invigorating them. Advent associated with CAR T-cell treatment marked a turning point in the advancement of cancer immunotherapy. This approach, which was first conceived in the latter part of the twentieth century, has been remarkably refined. Chimeric antigen receptors [CARs], which target particular proteins that are located on the surface of cancer cells, are expressed by T cells, a kind of immune cell that is modified as part related to CAR T-cell therapy. T cells might now identify and eliminate cancer cells with more efficiency, providing a tailored and focused treatment approach [14]. Cancer is a prerequisite for the creation of targeted medications. For a number of hematologic malignancies, such as lymphoma and leukemia, where conventional treatments might not be sufficient, CAR T-cell therapy has shown encouraging outcomes. The epidemiology of these cancers emphasizes the need for innovative solutions because the prevalence of these diseases is rising globally, creating a pressing demand for affordable and effective treatments^.^ Globally, the discipline of oncology is evolving because of CAR T-cell therapy. A larger patient group can now access this revolutionary medicine thanks to regulatory clearances made possible by clinical trials and successful applications. As technology advances, collaborations between academic institutions, pharmaceutical companies, and healthcare providers have expedited the assimilation of CAR T-cell therapy into conventional cancer care, offering renewed hope to patients who were previously facing bleak prognoses [14]. Developed nations, like the USA and Europe, have embraced this advancement, observing outstanding success stories and improvements in patient outcomes [15–17]. India has a high cancer rate, so the development of CAR T-cell therapy is a significant step forward in the fight against the disease. Allowing access to cutting-edge medications like CAR T-cell therapy may aid in fulfilling the medical needs that are not currently met for cancer patients nationwide. Nevertheless, issues with accessibility, infrastructure, and awareness continue to exist, highlighting the necessity of coordinated efforts to guarantee fair access and effective application of this revolutionary treatment [18].

From a theoretical idea to a potentially life-saving treatment, CAR T-cell therapy provides advanced remarkably throughout the past few years. Advances in genetic engineering, deeper comprehension of immune system components, and enhanced manufacturing methods have brought CAR T-cell therapy to the leading edge of cancer treatment. These recent advancements are significant because they have the potential to increase treatment efficacy and broaden the spectrum of tumors that can be treated with CAR T-cell therapy [19].

Immunotherapy utilizes the patient's immune system to identify and eliminate cancerous cells. Several cancer immunotherapies aim to improve immune cells’ potential to fight cancer by eliminating or improving their immune-suppressive state. Treatment by using CAR-T cell is an adoptive T-cell transfer [ACT] utilized in immunotherapy and has shown promising efficacy and long-term therapeutic response [20].

CAR-T cell therapy is a widely utilized form of cellular treatment. CAR-T cells are T cells that have undergone genetic modification to demonstrate a receptor intended for the detection and eradication of cancer. In the years 2020 and 2021, CD19–CAR-T therapies were authorized [21].

Many CAR-T researches related to cancer treatment have progressed to the stage of clinical trials [11].

We explore the advancing area of allogeneic hematopoietic stem cell transplantation and the initial results of CAR T-cell therapy for ALL. After the phase 2 ELIANA trial, which involved 79 pediatric and young adult patients with CD19 + R/R B cell ALL [median age: 11 years; age range: 3–24 years], the US FDA granted approval for sisa-cel, or sisalecleucel, marking it as the first CAR T-cell therapy for B-ALL [22]. The most prevalent primary brain tumor is Glioblastoma [9]. T cell therapy based on CAR- for hematologic malignancies and solid tumors, with preclinical results [23].

## Literature search and methodology

This review was performed by examining the literature published between 2019 and 2024, utilizing keywords like “CAR T-cell therapy,” “cancer immunotherapy,” “clinical trials,” and “hematological malignancies.” Primary databases used were PubMed, Google Scholar, and Web of Science. Articles were selected based on their relevance to CAR T-cell immunotherapy, clinical outcomes, limitations, and advancements in the field.

### Background of cancer immunotherapy

Because of his groundbreaking work, Dr. Coley is now referred to as the “Father of Cancer Immunotherapy”. But starting in the 1940s, applications of Coley's Toxins as a cancer treatment began to diminish, and by the 1960s, it had mostly stopped^24^. Gideon Gross and ZeligEshhar, two immunologists from the Weizmann Institute of Sciences in Israel's Department of Chemical Immunology, created the first T cell transformed with the chimeric molecule between 1989 and 1993 [6].

Immunotherapy hailed as a major advancement in cancer treatment, has its roots in discoveries made by physicians in the 1860s regarding tumor regression in erysipelas infection patients. Dr. William B. Coley’s revolutionary work with “Coley’s Toxins” in the 1890s, which produced remarkable outcomes in the treatment of cancer patients, expanded these discoveries. Subsequent discoveries in the twentieth century represented major turning points, including the invention of Bacillus Calmette-Guerin [BCG] immunotherapy and Raymond Pearl’s observation of a lower cancer incidence in patients with Mycobacterium TB. In the middle of the twentieth century, interferons and interleukin-2 [IL-2] showed promise as treatments, and by the late twentieth century, the FDA had approved them for use as cancer therapies. In 2018, the Nobel Prize was awarded for the discovery of immune checkpoint treatments, a breakthrough that changed the landscape of cancer therapy and resulted in FDA-approved antibodies aimed at CTLA-4, PD-1, and PD-L1. Tumor-infiltrating lymphocytes [TILs]-based adoptive transfer treatment has also demonstrated promise. Obstacles still exist, such as tumor immunogenicity and response rates, highlighting the continuous pursuit of boosting the effectiveness of cancer immunotherapy [20].

When researchers started looking at ways to alter T cells directed towards targeting cancer cells in the 1980s, the groundwork for CAR T-cell therapy was set. Israeli immunologist Zelig Eshhar and his colleagues first proposed the idea of modifying T cells using genetic techniques to produce chimeric antigen receptors (CARs) in 1989. Through the use of these CARs, T cells were capable of detecting and removing cancer cells without using the body’s normal immune systems. First-generation CAR T-cells were less effective as compared to later CAR T-cells because they only had one antigen recognition domain and were not as effective against solid tumors. Still, these initial experiments prepared the way for more advancements. The development of second-generation CARs in the 2000s, which included costimulatory domains like CD28 and 4-1BB and greatly increased sustained presence and efficacy of CAR T-cells was the breakthrough. Due to these advancements, the FDA approved Kymriah, the first CAR T-cell therapy, in 2017 for the treatment of relapsed or resistant B cell acute lymphoblastic leukemia (ALL). The significant success CAR T-cell therapies have shown in treating a variety of hematological malignancies, including Multiple myeloma and lymphoma have led to the approval of numerous CAR T-cell products targeting CD19 and BCMA since then [24, 25].

**Fig. 1:**
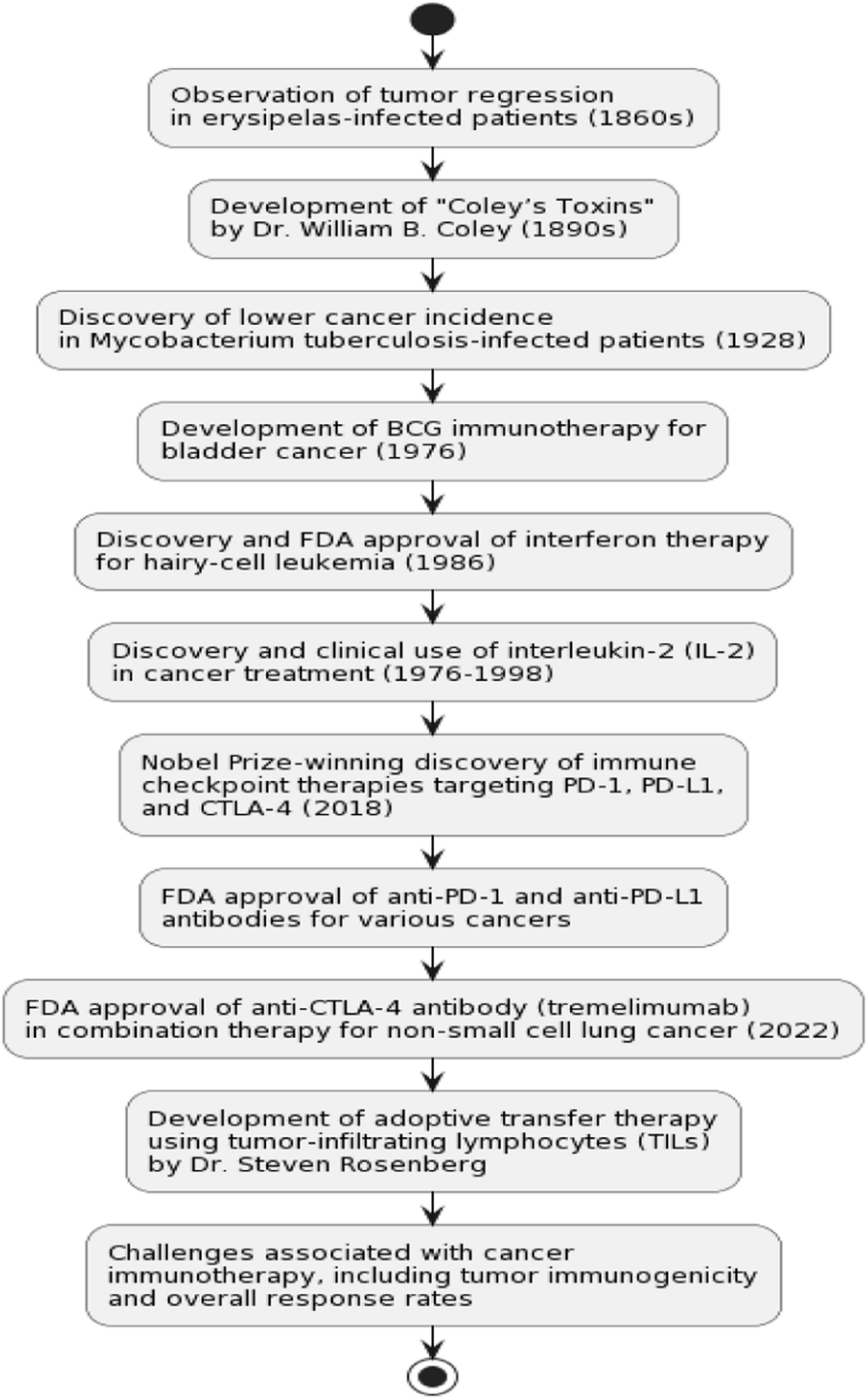
History of cancer immunotherapy

These pioneering studies extended efforts to harness the immune system’s potential to battle cancer and enabled the advancement of first-generation CARs. These early iterations only had one antigen-recognition domain and were primarily experimental. The first time CAR T-cells showed any real efficacy was with the development of second-generation CARs in the 2000s, which included costimulatory signals like CD28 and 4-1BB, particularly in hematologic malignancies. Because of the groundbreaking clinical successes, the FDA approved tisanecleucel (Kymriah), the initial CAR T-cell therapy, authorized in 2017 for the management of pediatric acute lymphoblastic leukemia (ALL). As of 2023, six CAR T-cell treatments have been authorized by the FDA making them an essential treatment for a range of cancers. With the goal of overcoming current challenges like cytokine release syndrome and expanding the utilization of CAR T-cells for non-blood cancers, yet the field is still at the beginning of its development [26] (as shown in Fig. 1).

### CAR T-cell therapy: evolution

The history of the allogeneic stem cell transplant procedure offers one of the leading as well as convincing arguments yet for immunotherapy’s ability to treat cancer [21]. Enhancing the efficacy and safety profile of the next generation of CAR-T cells is the aim of current engineering methods [12].

One cutting-edge immunotherapy method that is becoming more and more popular is the utilization of CAR-T cells to treat cancer. Single-chain variable fragment [scFv], which specifically and non-MHC-restrictedly identifies tumor antigens, is engineered into CAR-T cells. This portion is associated with transmembrane and intracellular signaling domains and originates from the variable domains of antibodies [23].

### Significance of recent advances

Clinical trials are evaluating the effectiveness of CAR-T cell therapies as initial treatments; even though the FDA has approved some medications for use as second and third-line treatments^12^.

### CAR T cell therapy: benefits

CAR-T cell therapy has several advantages, particularly in the mitigation of hematological malignancies. Its high specificity for cancer cells targets tumor-associated antigens (TAAs), minimizing the amount of off-target effects on healthy cells. Moreover, CAR-T cells’ ability to fight cancer is enhanced by their ability of cancer cells to avoid recognition by the immune system by functioning outside involving major histocompatibility complex (MHC) molecules. Two relapsed and resistant cancers, B cell acute lymphoblastic leukemia (ALL) and chronic lymphocytic leukemia (CLL), have shown remarkable responses to CAR-T therapy. It has occasionally produced long-lasting remissions, even for patients with few other options for treatment. The ability to tailor CAR-T therapy to each patient, resulting in a highly personalized treatment plan for each cancer, is one of the therapy’s primary benefits. Moreover, antigen escape—a technique by which cancer cells alter to avoid detection—is less likely when CAR-T cells are capable of recognizing several antigens. CAR-T cells incorporate co-stimulatory domains like CD28 and 4-1BB to increase T cell sustainability and effectiveness, which fortifies the T cells’ resistance to resistance mechanisms. When combined, these advantages turn CAR-T therapy into a ground-breaking therapeutic option for patients with hard-to-treat cancers [27].

## Fundamentals of CAR T-cell treatment

Treating several blood cancers accompanied by immunotherapy that uses T cells modified to bind to the CAR has demonstrated hitherto unheard-of results. Nonetheless, there are still some obstacles to be addressed for this novel therapeutic strategy to be therapeutically advantageous for solid tumors like lung cancer. With approximately 1.8 million deaths per year from cancer, lung cancer remains the leading cause of cancer-induced deaths across the globe. Identifying secure tumor-specific targets serves as a crucial limitation in developing CAR T-cell immunotherapy for lung cancer, which is why so many candidates have been, explored thus far [15].

T cells have been modified to produce synthetic receptors called CARs. This improves the cells' capacity to identify and destroy cancer cells. CARs integrate an antigen recognition domain, frequently generated from an antibody, with components of T cell activation. CAR-T cells show lethal effects including cell lysis and cytokine release when they come into contact with a target antigen in cancer cells. There is still more research to be done on this innovative immunotherapy approach's potential to treat many cancer types. The treatment of certain haematologic malignancies exhibited notable effectiveness with it. The concept of CARs offers a feasible route for more sophisticated immunotherapy and customized cancer care. Targeted therapy via T cell engineering: a potential cancer treatment strategy to be utilized in targeted therapy, T cells have to be genetically altered to express particular receptors, including chimeric antigen receptors [CARs], that allow them to find and eradicate cancer cells. Since this customized technique targets tumors precisely and effectively while causing the least amount of damage to healthy areas, it holds enormous potential for treating cancer. Patients’ T cells are collected and altered at the genetic level to express the specific receptors needed, then transformed cells are cultivated, and subsequently introduced into the patient’s body is reinfused with the modified cells. This ground-breaking method is transforming the way cancer is treated, resulting in more favorable prognoses and an improved quality of life projected for patients [20].

### Overview of immunotherapy

Over the past few decades, Targeted immunotherapies have significantly enhanced clinical outcomes in cancer treatment [28].

The use of targeted immunotherapies has markedly improved clinical results in cancer therapy. The use of NK cells in immunotherapy has gained increased attention in recent times. The process of CAR-T immunotherapy involves apheresis and the intricate expansion of immune cells derived from the patient’s own body [11]. The concept of immunotherapy was first applied in a clinical setting to cure cancer patients in the late 1800s using a modified toxin that became known as the Coley toxin [21].

### Engineering T-cells for targeted therapy

CARs, which provide T cells with the emergence of gene editing technologies, have led to the production of cytotoxic T cells that possess heightened and targeted capacity for killing. Furthermore, patients diagnosed with cancer who have gone through extensive pre-treatment are not able to donate enough normal T cells, which presents another obstacle to the production of CAR-T cells. NK cells can identify cancer cells depending on their human leukocyte antigen [HLA], unlike T cells. NK cells rarely result in significant toxicity. NK cells with CAR engineering can get beyond several obstacles that keep CAR-T treatment from being used more widely [11].

## Key components of CAR T-cells

The evolution of techniques utilized for gene editing has allowed for the insertion of CARs into cytotoxic T cells, giving T cells a more potent and targeted killing ability. Furthermore, patients with cancer who have undergone extensive pre-treatment are not able to donate enough normal T cells, which presents another obstacle to the production of T cells modified with CAR technology. NK cells can identify cancer cells depending on their HLA, unlike T cells. NK cells rarely result in significant toxicity. NK cells with CAR technology can get beyond several obstacles that keep CAR-T implementation [11]. NK cells to dosages that are medicinally meaningful. Feeder cells can significantly boost NK proliferation; nevertheless, they might be challenging to remove entirely from the culture, raising safety concerns [8].

A typical source of the extracellular domain, or the portion of an antibody that identifies antigens, is a single-chain variable fragment, or scFv. It adheres itself selectively to antigens on target cells’ surfaces, including cancer cells. Transmembrane Domain: This offers structural support and links the CAR to the T cellmembrane. Intracellular signaling domain: signaling motifs involve one or more that, upon binding of antigen, prompt T [11].

## Mechanism of action

On CAR-T cells, synthetic antigen receptors—called CARs—are created through engineering. These CARs are intended to target specific antibodies, including CD19, that appear on the surface of cancer cells in some blood cancers. When a CAR-T cell comes into contact with cancer cells expressing the target antigen, its surface attaches itself to the antigen, starting a signaling cascade that follows the CAR-T cell [14]. Once the CAR-T cell binds to the target antigen, it gets activated. This activation phase draws in multiple intracellular signaling molecules, which are phosphorylated and subsequently activate downstream pathways like PI3K/Akt and MAPK [29].

When these pathways are activated, the immune system’s protection against cancer cells is strengthened by the generation of cytokines and other effector molecules. The quick proliferation of activated CAR-T cells raises the total number of CAR-T cells. This proliferation is driven by cytokines, and interleukin-2 [IL-2], which are generated by other immune cells and CAR-T cells in response to CAR-T cell activation. More CAR-T cells allow for more accurate cancer cell targeting and removal from the body. After being triggered and growing, CAR-T cells use a variety of methods to kill tumor cells [30].

**Fig. 2:**
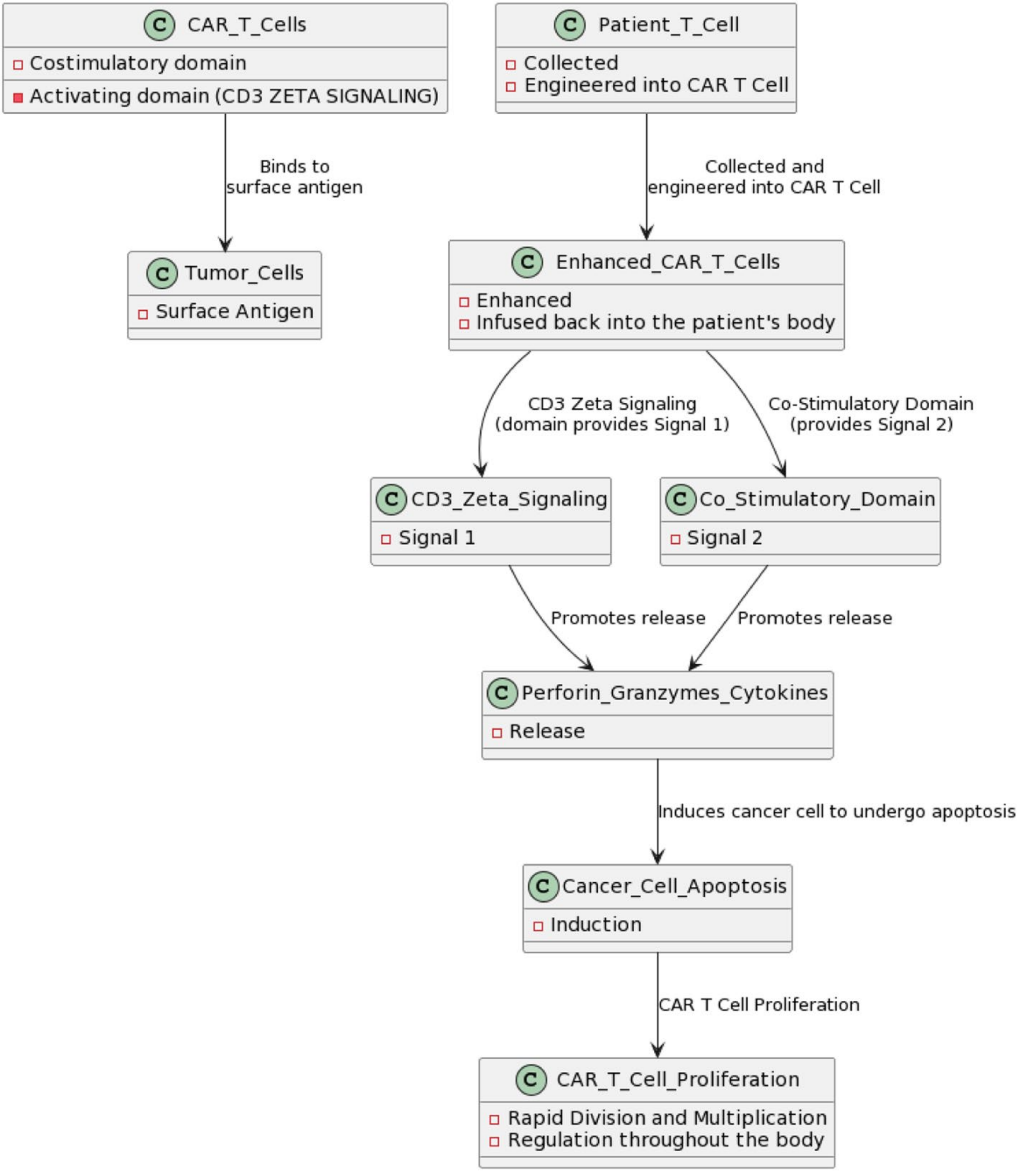
Disclosing the mechanisms of action of CAR-T cell therapy in treatment of cancer

One of these strategies is the production of cytotoxic substances, such as perforin and granzymes, which trigger apoptosis or apoptosis, in the target cancer cells. CAR-T cells can also engage in antibody-dependent cell-mediated cytotoxicity [ADCC] and cytokine-mediated death to increase their ability to destroy cancer cells. Since cancer cells may develop a defense against immune system recognition, It is vital for CAR-T cells to effectively recognize and specifically target cancer cells that present target antigens [31] (as shown in Fig. 2).

### Interaction with cancer cells

Romain et al. looked into the significance of T cell and tumor cells’ dynamic interactions to make discoveries that have practical applications correlation was discovered in the directed transfer of CD19-specific CAR-T cells and their versatility. Their findings demonstrated a connection between T cell movement and CD2 in T cells. Research on the dynamic CAR-T-tumor cell interactions is crucial because it enhances serial killing. The presence of CD2 in T cells is connected to CD58 expressed in lymphoma cells [32].

### Novel targets in CAR-T cell therapy: GPRC5D and CS1

#### CS1 as target in CAR-T cell therapy

Multiple myeloma (MM) cells express CS1 (SLAMF7), a glycoprotein that has shown promise as a target of CAR T-cell therapy, especially in cases of relapsed or refractory MM. Studies have demonstrated that elotuzumab and other CS1-targeted treatments are successful in treating patients with multiple sclerosis. To improve treatment results, CS1 combination therapy with other targets, such as BCMA, is currently being investigated. Significant clinical activity was observed in patients with relapsed or refractory multiple myeloma who received bispecific CS1-BCMA CAR-T cells, according to a recent study. In this trial, 81% of patients responded to the treatment, and 38% of patients experienced a strict complete response. Through dual targeting of CS1 and BCMA, myeloma cells are attacked from two different angles, which decreases antigen escape and increases treatment efficacy. Strong anti-myeloma activity was present, but there were also potential side effects, like cytokine release syndrome (CRS) and hematological toxicities, that could be managed with appropriate care [33].

#### GPRC5D as target in CAR-T cell therapy

The G protein-coupled receptor class C group 5 member D (GPRC5D) has recently been recognized as a target for CAR T-cell therapy. Its main area of study is its potential to treat multiple myeloma, especially in individuals who have experienced a relapse following BCMA-targeted therapies. Since GPRC5D is only expressed in myeloma cells and some keratinized tissues, it is a prospective target with little off-tumor consequences. Patients who had experienced significant pretreatment, including BCMA CAR-T therapy, showed a 71% response rate in a recent phase 1 trial employing MCARH109, CAR T-cell therapy targeted at GPRC5D. The therapy exhibited an overall acceptable safety profile; however, the most common side effects were cytokine release syndrome and hematologic toxicities [34].

## Clinical applications

With the groundbreaking discovery of CAR-T cell therapy, individuals who have exhausted all other options for conventional cancer treatment now have hope again. Numerous CAR-T cell treatments that target particular tumors have been authorized for implementation in clinical practice, and numerous ongoing studies are testing the efficacy of these treatments in a range of patient populations [30].

Among its most notable achievements is the acceptance of related to CAR-T cell therapy to treat specific blood-related malignancies, especially B cell malignancies. Tisagenlecleucel [Kymriah] and axicabtageneciloleucel [Yescarta], two CAR-T cell therapies, have obtained authorization from the FDA and EMA to address diffuse large B cell lymphoma (DLBCL) and relapsed or refractory B cell acute lymphoblastic leukemia (ALL). The medications have shown remarkable response rates and enduring advantages [28].

### Authorized CAR T-cell treatments

Investigating the function of metabolism in the immune system and malignancies has become easier because of modern technologies like organoid technology [35]. The US FDA has approved Kymriah [Novartis], Yescarta [Gilead], Tecartus [Gilead], Breyanzi [Bristol Myers Squibb], Abecma [Bristol Myers Squibb and Bluebird Bio], and Carvykti [Legend and Janssen] as the six CAR-T products used in the management of hematological cancers [11]. Most cancer patients’ tumor-free survival rate following standard therapy, despite considerable improvement [28].

Both preclinical investigations and early clinical findings have highlighted the significant promise of CAR-NK cells as ready-to-use cancer therapies. The evolution of the CAR methodology has largely been propelled by advancements in CAR-T cell therapies [11].

### In-progress clinical trials

Nowadays, a minor count of CAR-T cells is creating profitable brands [11].

Concerning multiple myeloma, B cell acute lymphoblastic leukemia [ALL], and relapsed and refractory [R/R], one of the most notable innovative therapy possibilities is chimeric antigen receptor [CAR]-T cells for B cell non-Hodgkin lymphomas [B-NHLs]. Future methodologies may incorporate direct analysis of single-cell metabolites and the development of reliable techniques for isolating individual cells while minimizing metabolic disruptions [35]. The application of [CAR] T-cells in treating hematologic malignancies, alongside numerous preclinical studies suggesting their promise for solid tumors [23].

CAR-T cell therapies have demonstrated encouraging outcomes in multiple clinical trials for the treatment of various lymphomas. During the ZUMA-5 trial, individuals with follicular lymphoma were assessed using Axi-cel, which targets the CD19 antigen. A 79% response rate was shown, and 54% of patients experienced complete remission, or being free of cancer for at least 3 years. 6% of patients experienced severe cytokine release syndrome (CRS), a possible adverse effect. Another CD19-targeting treatment for follicular lymphoma, tira-cel, demonstrated a 68% response rate in the ELARA trial, with 57% of patients continuing to be cancer-free after 2 years and 88% surviving for a minimum of 2 years. Notably, no patients experienced severe CRS. In the Liso-cel TRANSCEND trial, the response rate for follicular lymphoma was 94%, with 81% of patients in remission for at least 1 year, and only 1% developed severe CRS. For marginal zone lymphoma, Axi-cel showed a 63% response rate and 70% of patients survived for at least 2 years, with severe CRS reported in 9% of patients. Finally, TanCAR7, which affects both CD19 and CD20 antigen targets demonstrated a 70% response rate in cases of B cell non-Hodgkin lymphoma, accompanied by severe CRS occurring in 10% of patients [36] (as shown in Table 1).

**Table 1:** Ongoing clinical trials

CAR-T product	Target antigen(s)	Cancer type	Trial name/phase	Overall response rate (ORR)	Complete response (CR)	Progression-free survival (PFS)	Release syndrome (CRS, Grade ≥ 3)
Axi-cel	CD19	Follicular lymphoma (FL)	ZUMA-5	79%	54% (3-year PFS)	75% (3-year OS)	6%
Tisa-cel	CD19	Follicular lymphoma (FL)	ELARA	68%	57% (2-year PFS)	88% (2-year OS)	0%
Liso-cel	CD19	Follicular lymphoma (FL)	TRANSCEND	94.1%	81% (1-year PFS)	–	1%
Axi-cel	CD19	Marginal zone lymphoma	ZUMA-5	63%	70% (2-year OS)	–	9%
TanCAR7	CD19/CD20	B-NHL	–	70%	–	–	10%

## Challenges and limitations

Certain toxicities are linked to cancer immunotherapies. The effectiveness of these drugs can be increased by developing grade-based treatment plans for toxicity syndromes along with scoring levels. Nevertheless, some noteworthy challenges still exist, such as prohibitive expenses, restricted clinical availability, resistance to treatment, recurrence, and unfavorable results. One of these challenges that can hinder the capacity of CAR-T cells to identify and efficiently target cancer cells is tumor antigen loss. Another is heterogeneity. Furthermore, CAR-T cells often have difficulty encroaching on solid tumors due to their distinct microenvironments. The limited survival and endurance of CAR-T cells related to these immunosuppressive environments further limits their therapeutic potential. Moreover, there are several side effects related to CAR-T cell therapy, such as neurotoxicity, cytokine release syndrome (CRS), on-target off-tumor toxicity (OTOT), and graft-versus-host disease (GvHD), all of which pose serious difficulties for patient care [1, 4].

A condition of localized neurotoxicity, which we have named TIANhas resulted through clinical and preclinical research using cell therapies for brain cancers. This illness is different from the systemic CRS and ICANS toxicity syndromes [2009]. Another emerging danger to CAR therapies is trophocytosis, a process that results in the transfer of cell membranes expanding from target cells to immune cells and the fratricidal eradication of CAR cells [8].

Cytokine release syndrome (CRS) and immune effector cell-associated neurotoxicity syndrome (ICANS) rank as two of the most common adverse effects [23]. The utilization of CAR-T cells has revolutionized the way cancer patients are treated, particularly those with hematologic malignancies that have relapsed or shown no improvement with previous treatments. However, there are still a lot of barriers and limitations, like CNS injury, antigen escape mechanisms, and CRS. To advance the field and improve patient outcomes, it is imperative to recognize and deal with these challenges. The activation of CAR-T cells, which express cytokines such as TNF-α, interleukin-6 [IL-6], and interferon-gamma [IFN-γ], results in the systemic inflammatory response, or CRS. Fever, hypotension, multiorgan failure, and capillary leak syndrome are examples of severe CRS symptoms. It can be very difficult to treat any of these conditions [13].

CAR-T cell activation, which triggers the release of cytokines like TNF-α, interleukin-6 (IL-6), and interferon-gamma (IFN-γ), leads to a systemic inflammatory response known as cytokine release syndrome (CRS). Fever, hypotension, multiorgan failure, and capillary leak syndrome are examples of severe CRS symptoms. It can be very difficult to treat any of these conditions. Researchers are looking into new ways to lower CRS without sacrificing anti-tumor activity. Cytokine scavenging agents and suicide switch incorporation into CAR-T cells through genetic alteration is one example of these strategies [19].

The advancement of CAR-T therapies for solid tumors is hindered by the limited availability of specific surface targets on tumor cells. Nevertheless, there have been some encouraging pre-clinical and clinical trial developments with CAR-T cell therapy for solid tumors that target distinct cell surface antigens [37].

Prognoses have improved and many patients have been spared from aggressive cancers thanks to the outstanding results of CAR T cell treatments. However, an increasing amount of clinical experience is routinely highlighting their shortcomings. There are various barriers that impede the development of novel and enhanced treatments in addition to those that are currently approved. These difficulties include antigen escape events, low persistence of CAR T cells, low tumor-killing efficacy (especially when removing solid tumors), and high toxicity, which can result in severe CRS and neurotoxic side effects [38].

### Antigen escape

One of the most significant challenges of CAR-T cell therapy is tumor resistance to CAR constructs that target a single antigen. Although CAR-T cells aimed at a single antigen can initially produce high response rates, many patients treated with these cells possess malignant cells that show partial or complete loss of target antigen expression. This phenomenon is known as antigen escape [39].

### Adverse effects of CAR T-cell therapy

ICANS is among several common side effects associated with CAR T-cell therapy. It is thought that the impairment of the blood–brain barrier and the activation of brain endothelial cells during inflammatory responses are two key factors contributing to the onset and progression of ICANS [4].

### Cytokine release syndrome [CRS]

Cytokine release syndrome (CRS) is a severe and potentially life-threatening side effect of CAR-T cell therapy, resulting from the rapid immune activation that occurs after CAR-T cell infusion. When CAR-T cells detect tumor antigens, they become activated and release large quantities of cytokines, including TNF-α, IFN-γ, and IL-6. This cytokine storm results in fever, myalgia, and exhaustion as well as widespread systemic inflammation. If CRS is not treated, it can worsen and cause shock, hypotension, capillary leakage, and multiorgan dysfunction. Since IL-6 is thought to be a major cause of CRS, treatments like the IL-6 receptor antagonist tocilizumab are frequently employed to treat severe cases. Effective dosing and cytokine management strategies are essential because high disease burden and elevated CAR-T cell doses are associated with an increased risk of CRS [40, 41].

Tachycardia, tachypnea, fevers, rigors, and myalgias are among the milder signs and symptoms of CRS. On the other hand, more severe symptoms may include coagulopathy, liver damage, hypoxia, arrhythmias, renal failure, and malfunction of other essential organs [42].

Not only does CAR-T therapy have remarkable response rates, but it was soon discovered that there is a chance of potentially dangerous systemic toxicities, which are typified by strong and widespread immune system stimulation [43].

### Syndrome of cytokine release

CRS is the most common side effect of CAR-T cell therapy due to the release of inflammatory cytokines and chemokines when CAR-T cells bind to target antigens. Research indicates that CAR-T cells activate gastrin E (GSDME) to induce cell death and contribute to CRS by releasing significant quantities of granzyme B and perforin. Fever may arise within hours, days, or even weeks following cell infusion. Deterioration is seen in hypotension, capillary leakage, and organ dysfunction. The most important part of CRS care is limiting the use of CAR-T-cell therapy to tolerant individuals. Direct targeting and non-specific immunosuppression are two supportive therapies that should be given to patients and CAR-T cell products. Early tocilizumab and/or corticosteroid treatment reduces grade 3 or higher CRS, according to studies. As CAR-T-cell therapy becomes more widespread, practitioners will gather knowledge to standardize diagnostic and therapeutic procedures for CRS, enhancing CAR-T-cell safety [44].

### Neurological toxicities

Fewer people are affected by ICANS, which results in a variety of non-specific neurologic toxicities. Neurotoxicity of immunological effector cells. ICANS represents another typical side effect of CAR-T cell therapy that can occur with or without the presence of CRS. Neurotoxicity was observed in 37.2% of individuals with hematologic malignancies after receiving CAR-T cell therapy. Consequences from progressive aphasia include cerebral edema, convulsions, paralysis, and coma. Fasting, hydration, nutritional support, and neurological testing are all part of the ICANS therapy regimen. Thus, ICANS/CRS-related fever, inflammatory cytokines, and biomarkers decreased rapidly. Early diagnosis and prevention are crucial for managing ICANS, a progressive CAR-T-cell treatment adverse effect [43].

Moreover, by recognizing and cleaving healthy cells expressing targeted antigens, CAR-T-cell treatment often causes severe on-target, off-tumor [OTOT] toxicity, toxicity that has been connected to toxicity in solid tumor clinical trials. TLS, GVHD, DIC, and phagocytic syndrome are side effects of CAR-T-cell treatment [44].

### On-target off-tumor toxicity (OTOT)

On-target off-tumor toxicity (OTOT) is a condition caused by CAR-T cells that target antigens present in both tumor cells and healthy tissues. Because CAR-T cells are engineered to recognize specific antigens, it is possible that they will inadvertently target healthy tissues that express the same antigen, which could be harmful. The fact that healthy cells share a significant number of target antigens found in solid tumors makes OTOT a significant obstacle to the wider application of CAR-T cell therapy. The symptoms of OTOT may differ based on the tissue affected, but they may include toxicity and major organ damage. Two recommended strategies to mitigate OTOT are to design CAR-T cells with dual targeting systems, which allow for more precise tumor-specific antigen recognition, or to use safety switches to deactivate CAR-T cells when off-target effects are identified [41].

### Graft-versus-host disease (GvHD)

Graft-versus-host disease (GvHD) is a recognized adverse effect of CAR-T cell therapy that is more prevalent when CAR-T cells from a donor are used. Donor T cells attack the patient’s healthy tissues, causing harm to the liver, gastrointestinal tract, and skin. This condition is known as gene-versus-host disease (GvHD). Patients with previously undergone hematopoietic cell transplantation (HCT) are often affected by this condition, which is characterized by the donor attacking the host’s immune system. Using autologous CAR-T cells, which come from a patient’s T cells, lowers the risk of GvHD. However, in light of the growing interest in developing allogeneic CAR-T therapies, approaches to reduce GvHD, like T cell receptor modification and selective tumor antigen targeting, are being investigated [45].

### Neurocognitive therapy and movement

Treatment-emergent adverse events (MNTs) related to movement and neurocognition are examples of non-ICANS neurologic toxicity. Recently, BCMA CAR-T cell patients having multiple myeloma have reported MNTs. MNT resembles Parkinsonian illness, although neuropathology and BCMA expression in the brain parenchyma does not indicate off-target, on-target toxicity [43].

### Other safety concerns

Healthcare professionals must be aware of the potential risks associated with CAR T cell therapies during the days following infusion. These risks include unpredictable off-target toxicity, anaphylaxis, and graft-versus-host disease (particularly with allogeneic treatments). Additionally, there is a lesser but theoretical concern regarding viral insertional oncogenesis when employing viral vectors [38].

## Innovations and advancements

Many strategies, like CAR subunit dimerizing agents, CAR T-cells connected to tiny-molecular connector agents with on/off switches, downstream CAR signaling inhibitors, inhibitors of protease to regulate CAR protein expression, also constructed enhanced CAR T-cells that can generate factors, have been employed by studies to counteract the systemic cytokine toxicities. Since toxicity arises from going beyond the therapeutic window, The CAR T-cell needs to remain within it to have therapeutic benefits [6].

Deciphering and advancing strategies:

- 1. Combination therapy.
- 2. Engineered CAR T-cells.
- 3. Directing CAR T-cells to specific areas.

The discipline of immunotherapy has advanced significantly In recent times, particularly with the development of gene editing and CAR-T (chimeric antigen receptor T cell) technology. Millions of people around the world now have hope thanks to these ground-breaking methods that have transformed the way that many diseases, including cancer and genetic abnormalities, are treated. The objective of CAR-T cell therapy is to modify T cells so they can more efficiently target and combat cancer cells in the body. In cases when conventional treatments have failed, this individualized method has shown exceptional success in treating some kinds of leukemia and lymphoma. Notwithstanding its efficacy, CAR-T therapy has encountered obstacles like restricted response durability and unfavorable side effects. Researchers are concentrating on developing next-generation CAR-T cell treatments with improved characteristics and functions to get beyond these obstacles. One possible approach is to create “armored” CAR-T cells, which are tailored to express additional proteins or chemicals to boost their effectiveness and persistence within the body. Suicide genes for safety control, cytokines to boost immune responses, and checkpoint inhibitors to reverse immunosuppression in the tumor microenvironment are a few examples of these improvements [15].

Furthermore, advancements and breakthroughs in genome editing technologies, particularly CRISPR-Cas9, have expanded the possibilities for optimizing CAR-related applications. Researchers can improve T cell targeting specificity, avoid immunological rejection, and increase their overall therapeutic potential by precisely modifying their genomes using CRISPR-based methods. Additionally, gene editing techniques might be employed to get around the difficulties involved in producing CAR-T cells on a large scale, which will lower the cost and increase the accessibility of these treatments [19].

Gene editing techniques have great potential for treating a variety of genetic problems, not just cancer. Gene editing therapies are being used to treat diseases including sickle cell disease and Duchenne muscular dystrophy which were originally believed to be incurable. These therapies aim to fix the underlying genetic abnormalities causing these conditions. Through careful manipulation of the impacted cells’ DNA sequence, scientists may be able to offer sustained or possibly irreversible alleviation from these crippling ailments. Furthermore, there are still major obstacles in the way of utilizing gene editing and using CAR-T cells to their maximum potential, including cost and accessibility. Although many patients find hope in these treatments, their general adoption is limited by their high costs and intricate production methods. To create long-lasting solutions that guarantee fair access to these life-saving treatments, researchers, healthcare professionals, legislators, and industry stakeholders must work together to address these obstacles [46].

### Advanced CAR T-cell therapies

Even though CAR-T treatment has advanced significantly, much more research is needed before it can be used to treat a larger range of tumor types. With patient data, single-cell technologies will undoubtedly offer a crucial understanding of the dynamics between cancer cells, CAR-T cells, and other immune groups. A thorough discussion is necessary for many fundamental questions. Single-cell techniques would effectively gather this data, shaping the development of CAR-T therapy cells and other endogenous immune systems [11].

### Personalized CAR T-cell approaches

The treatment of CAR-T for different kinds of cancer is shown in Table 2.

**Table 2:** Treatment of CAR-T for different kinds of cancer

Hematological malignancies	• T lymphocytes have been crucial in eliminating hematological malignancies for many years, as demonstrated by the positive outcomes of allogeneic hematopoietic stem cell transplants • T cells were able to make contact with targets by using a single-chain variable fragment derived from an antibody [scvf] linked with internal T cell signaling domains. This allowed the T cells to get past HLA barriers and broaden their repertoire of tumor-recognition antigens • CAR-T cells were initially used to treat HIV patients, but have since acquired popularity in treating hematological cancers • Another significant benefit of hematology • Accessibility of cancers. Intravenous CAR-T cells interact better with blood-circulating tumor cells. How co-stimulation aids CAR-T cell proliferation in vivo within the human immune system [47]
Lymphoma	• Conventionally, human lymphomas are divided into two categories: non-Hodgkin and Hodgkin (NHL and HL) • A wide range of lymphoid malignancies, known as HLs, are brought on by B, T, or NK cell clonal expansions at various phases of development • While T and NK lymphomas can develop during any stage of normal lymphopoiesis, B cell lymphomas originate from B cells in the germinal center or post-germinal center stages • All lymphoma CARs are based on scFvs, with efficiency varying based on CAR affinity and antigenic epitope characteristics • Numerous studies back up the utilization of CAR-T cells to treat lymphoma, but they also point out two issues • • The technique is less effective for lymphoma than for acute B cell leukemia, and treatment-related toxicity may be fatal • • Enhancing CAR T cell safety and therapeutic efficacy is essential for successful therapy [48]
Pancreatic cancer	• Pancreatic cancer [PC] ranks 12th in prevalence and 7th in cancer-related deaths worldwide • Age, sex, tobacco use, obesity, high blood glucose levels, insulin resistance, type 2 diabetes, inflammation of the pancreas, and genetics are among the risk factors for PC • CAR-T cells that recognize PD-1 effectively eliminate PC cells [49]

## Emerging trends in immunotherapy

Over the years, several therapies have been developed to treat hematological malignancies, however, they continue to cause cancer deaths globally. Stem cell transplantation, chemotherapy, along radiation therapy are the primary treatments for hematological malignancies. Additionally, understanding malignant cell-immune system cell interactions has held promise for immunotherapy research. Immunotherapy was once a promising topic, but it has now revolutionized cancer treatment. Hematological malignancies can benefit from the immunotherapeutic treatment of chimeric antigen receptor [CAR] T-cell therapy. Tumor-associated antigens, or TAAs, are recognized with the help of genetically altered CAR-T cells which then activate T cells lacking MHC molecules. Immunotherapy using CAR-T cells for hematological malignancies has progressed. Although CAR-T-cell therapy has many advantages, some patients have experienced significant toxicities and unfavorable results. A few CAR-T cell treatments had setbacks and relapses. Therefore, additional study is required to advance this emerging immunotherapy method. Significant advancements in the treatment of hematological malignancies have been achieved with CAR-T-cell therapy. Increased longevity and efficacy of CAR-T cells in patients and decreased adverse effects are the primary issues with this treatment method. The major treatment medications corticosteroids and tocilizumab can also reduce CAR-T-cell therapy side effects. Using B cells supported by preclinical data to eliminate CAR-T cells through anti-CD19 CAR-T-cell mediation is one of the main strategies [50].

## CAR-T cell therapy beyond oncology

### 1. Asthma

The immune response dominance is a characteristic of asthma and allergic disorders [ADs]. CAR-T cells could potentially target IgE produced by B cells, which is crucial in the pathophysiology of allergic diseases (ADs). The binding of IgE to its receptor, FcεRI—found on mast cells, eosinophils, and basophils—initiates allergic reactions by causing degranulation and the subsequent release of inflammatory mediators.

- a. Targets of CAR-T lymphocytes include memory B cells expressing transmembrane IgE, germinal center B cells, plasma blasts, and plasma cells.
- b.The origins of allergic asthma are linked to an overload of Th2 cell-dominated reactions to allergens, which cause hyper-reactivity, reversible obstruction, and airway inflammation. It is also related to a minimum number of Tregs and limited immunosuppressive activity.
- c. The results showed that there was increased expression of Th2 cytokines, inhibition of functional CAR-T cells, and IgE specific to allergens in addition to airway hyperreactivity, eosinophilic airway inflammation, and increased mucus production [51].

### 2. Contagious illness

- a. CD8 + T cells are appealing CAR-Ts for infectious diseases because they eliminate invasive substances and cells. In their study, Krebs et al. created and assessed CAR-T cells featuring S domain receptors (S-CARs) for all three HBV envelope protein sequences (S, M, and L). This approach led to the presentation of HBV surface antigen (HBsAg) on the outer membrane of infected cells.
- b. Anti-HBs-G4m CAR-T cells can detect HBV-positive cells and HBsAg particles in vitro, and they are capable of reducing HBV-DNA and HBsAg levels in vivo. For patients suffering from end-stage chronic hepatitis C virus (HCV) who do not respond to treatment, liver transplantation may be considered.
- c. CAR-T cells displayed significant cytotoxicity against HCV/E2 in cells infected with HCV. HCMV infection and reactivation have been major causes of mortality in patients following primary organ and hematopoietic stem cell transplantation. Professor et al. sought to redirect T cells to HCMV glycoprotein B (gB), but anti-gB CAR-T cells did not successfully lyse infected cells in vitro, even after being activated. Regardless of their cytotoxic effector actions, stimulated CAR-T cells against gB reduce HCMV proliferation through IFNγ and TNFα [51].

### 3. HIV

- a.Combination antiretroviral therapy (CAR-T) can reduce HIV-1 replication, but eradicating the infected cells' latent reservoir is still a major problem.
- b. The gp120 segment of the HIV envelope (Env) glycoprotein on HIV-infected cells is believed to be the target of anti-HIV-1 CAR-T therapy. As such, CAR-T therapy for HIV-1 represents a promising new treatment strategy, similar to its application in oncology. Recently, two phase I clinical trials are recruiting participants to assess the safety and effectiveness of HIV CAR-T cell therapy. NCT03240328 trial is enrolling HIV patients whose plasma viral load has decreased following the initiation of combined antiretroviral therapy (cART), aiming to eradicate HIV reservoirs and enhance HIV-specific immune responses. Meanwhile, the NCT03980691 trial is exploring the combination of chidamide alongside CAR-T or TCR-T cell therapies designed to target HIV-1 latent reservoirs. The unmentioned NCT04648046 trial will assess the biosafety and specificity of CAR-T cells against gp120 [51].

### 4. COVID-19

COVID-19, which is triggered by the severe acute respiratory syndrome coronavirus, is a non-contagious infection. Researchers are examining CAR-transduced immune cells as a potential therapeutic option for targeting cells infected by the virus [51].

## Future perspectives

When chemotherapy fails, chimeric antigen receptor [CAR] T cell immunotherapy has helped certain cancer patients. Better CAR-T cell design and delivery could lead to a cure for many cancer patients. CAR-T cells are too dangerous, expensive, or inaccessible for most blood cancer and solid malignancy patients. Commercially and biologically, CAR technology is powerful. Studies are looking into the chronic impacts of CAR-T cells, which were administered to over 1000 patients in the USA. Blood malignancies that express CD19 react to CAR-T cell treatment best, for a number of well-established reasons. When applying CAR-T cell therapy for different diseases, new strategies are being explored for CD19-expressing conditions to prevent antigen-negative relapse, enhance tumor-killing effectiveness, boost CAR-T cell persistence, and manage activity and toxicity [51].

CAR-T cells have revolutionized the way that B cell acute lymphoblastic leukemia is managed. CAR-T cells are useful for treating a variety of hematologic malignancies; new CARs and combinations are emerging; and new challenges are emerging. However, ineffective CAR-T cell growth and longevity, in addition to regional inhibitory factors are frequently cited as the main reasons for inadequate response durability after CAR-T cell therapy. In a period spanning 2017 to 2022, the FDA granted approval for six CAR-T cell products, which illustrates how technology has changed over the previous 30 years. CAR-based therapy is being expanded in research to treat more solid tumors. The data obtained from in vitro experiments proved that CAR-T cells were properly generated [49].

The three primary areas where CAR-T therapies need to be improved are:

- a. To address Targeted toxicity, unintended effects on non-tumor tissues, and tumor variability, identify appropriate molecular targets.
- b. Ensure active, non-exhausted CAR-T cell survival in PC.
- c. Finding and removing tumor Cells that induce immunosuppression, such as Treg lymphocytes, TAMs, and MDSCs [49].

## Conclusion

In conclusion, pertaining to CAR T cell therapy from laboratory inception to life-saving application in oncology represents a remarkable stride in cancer therapy. By utilizing the immune system to fight cancer, the development of cancer immunotherapy—especially with the introduction of CAR T-cell therapy—has completely changed the way that cancer is addressed. The safety aspects and therapeutic effectiveness of CAR T-cell therapy have been greatly improved by recent developments, opening the door for its widespread clinical application.

Fundamental principles underlying CAR T-cell treatment, including engineering regarding T cells for targeted therapy, have laid the groundwork for its success. Key components of CAR T-cells, alongside elucidation regarding their mechanisms of action, have provided invaluable insights into their clinical applications. Validated CAR T-cell therapies and active clinical trials highlight the growing momentum in translating research findings into tangible patient benefits.

Even with significant advancements, there are still obstacles and restrictions, such as immunological-related toxins and neurological issues. Ongoing advancements in CAR T-cell therapies of the next generation, however, present encouraging ways to tackle these issues. Moreover, the expansion of CAR T-cell therapy outside of the cancer field highlights its adaptability and potential for treating a variety of illnesses.

The future of CAR T-cell therapy is extremely promising. Ongoing studies focused on tumor biology, the design of CAR T-cells, and the optimization of treatment protocols should further enhance therapeutic outcomes. With continued collaborative research, CAR T-cell therapy is poised to transform cancer treatment and provide hope to many patients globally.

## Data Availability

Data and materials are available upon request.

## Abbreviations

CAR-T: Chimeric antigen receptor tumor
scFv: Single chain variable fragment
FDA: Food and Drug Administration
NK cells: Natural killer cells
T cells: Tumor cells
mIgE: Transmembrane form of IgE
ACT: Adoptive T cells transfer
USA: United States of America
ALL: Acute lymphoblastic leukemia
BCG: Bacillus calemetteguerine
IL-2: Interleukin 2
TILs: Tumor-infiltrating lymphocytes
GB: Glycoprotein B
ILCs: Innate lymphoid cells
HLA: Human lymphoid antigen
ADCC: Antibody-dependent cell-mediated cytotoxicity.
DLBCL: Diffuse large B cell lymphoma
R/R: Replased and refractory
B-NHLs: B cells non-Hodgkin lymphomas
TNF alpha: Tumor necrosis factor-alpha
CRS: Cytokine release syndrome
ICANS: Immune effector cell associated neurotoxicity syndrome
CRF: Chronic renal failure
TAAs: Tumor-associated antigen
ADs: Allergic diseases
IgE: Immunoglobin
HCMV: Human cytomegalovirus
HIV: Human immunovirus
AEs: Adverse events
CART: Combination antiretroviral therapy
GBM: Glioblastoma
MHC: Major histocompatibility complex

## References

[1] Maalej KM, Merhi M, Inchakalody VP, et al. CAR-cell therapy in the era of solid tumor treatment: current challenges and emerging therapeutic advances. Mol Cancer. 2023;22(1):1–54. 10.1186/s12943-023-01723-z. 36717905 10.1186/s12943-023-01723-z PMC9885707

[2] DaeiSorkhabi A, Mohamed Khosroshahi L, Sarkesh A, et al. The current landscape of CAR T-cell therapy for solid tumors: Mechanisms, research progress, challenges, and counterstrategies. Front Immunol. 2023;14:91113882. 10.3389/fimmu.2023.1113882. 10.3389/fimmu.2023.1113882 PMC10067596 37020537

[3] Abbasi S, Totmaj MA, Abbasi M, Hajazimian S, Goleij P, Behroozi J, Shademan B, Isazadeh A, Baradaran B. Chimeric antigen receptor T (CAR‐T) cells: novel cell therapy for hematological malignancies. Cancer med.. 12(7), 7844–7858. 10.1002/cam4.5551. 10.1002/cam4.5551 PMC10134288 36583504

[4] Huang S, Wang X, Wang Y, Wang Y, Fang C, Wang Y, Chen S, Chen R, Lei T, Zhang Y, Xu X. Deciphering and advancing CAR T-cell therapy with single-cell sequencing technologies. Mol Cancer. 2023;22(1):1–25. 10.1186/s12943-023-01783-1. 37149643 10.1186/s12943-023-01783-1 PMC10163813

[5] Cappell KM, Kochenderfer JN. Long-term outcomes following CAR T cell therapy: what we know so far. Nat Rev Clin Oncol. 2023;20:359–71. 10.1038/s41571-023-00754-1. 37055515 10.1038/s41571-023-00754-1 PMC10100620

[6] Huang Z, Chavda VP, Bezbaruah R, Dhamne H, Yang DH, Zhao HB. CAR T-cell therapy for the management of mantle cell lymphoma. Mol Cancer. 2023;22(1):1–9. 10.1186/s12943-023-01755-5. 37004047 10.1186/s12943-023-01755-5 PMC10064560

[7] Chohan KL, Siegler EL, Kenderian SS. CAR-T cell therapy: the efficacy and toxicity balance. current hematologic malignancy reports. 2023;18(2):9–18. 10.1007/s11899-023-00687-7. 10.1007/s11899-023-00687-7 PMC10505056 36763238

[8] Kilgour MK, Bastin DJ, Lee SH, Ardolino M, McComb S, Visram A. Advancements in CAR-NK therapy: lessons to be learned from CAR-T therapy. Front. Immunol. 2023;.1–10. 10.3389/fimmu.2023.1166038. 10.3389/fimmu.2023.1166038 PMC10187144 37205115

[9] Majumder A. Evolving CAR-T-cell therapy for cancer treatment: from scientific discovery to cures. Cancers. 2023;16(1):1–28. 10.3390/cancers16010039. 38201467 10.3390/cancers16010039 PMC10777914

[10] Luksik AS, Yazigi E, Shah P, Jackson CM. CAR T cell therapy in glioblastoma: overcoming challenges related to antigen expression. Cancers. 2023;15(5):1–18. 10.3390/cancers15051414. 10.3390/cancers15051414 PMC10000604 36900205

[11] Zhang Y, Zhou W, Yang J, Yang J, Wang W. Chimeric antigen receptor engineered natural killer cells for cancer therapy. Exp Hematol Oncol. 2023;12(1):1–30. 10.1186/s40164-023-00431-0. 37563648 10.1186/s40164-023-00431-0 PMC10413722

[12] Tang L, Huang ZP, Mei H, Hu Y. Insights gained from single-cell analysis of chimeric antigen receptor T-cell immunotherapy in cancer. Mil Med Res. 2023;10(1):1–33. 10.1186/s40779-023-00486-4. 37941075 10.1186/s40779-023-00486-4 PMC10631149

[13] Noh JY, Seo H, Lee J, Jung H. Immunotherapy in hematologic malignancies: emerging therapies and novel approaches. Int J Mol Sci. 2020;21(21):1–23. 10.3390/ijms21218000. 10.3390/ijms21218000 PMC7663624 33121189

[14] Wudhikarn K, Perales MA. Infectious complications, immune reconstitution, and infection prophylaxis after CD19 chimeric antigen receptor T-cell therapy. Bone Marrow Transplant. 2022;57(10):1477–88. 10.1038/s41409-022-01756-w. 35840746 10.1038/s41409-022-01756-w PMC9285870

[15] Bupha-Intr O, Haeusler G, Chee L, Thursky K, Slavin M, Teh B. CAR-T cell therapy and infection: a review. Expert Rev Anti Infect Ther. 2021;19(6):749–58. 10.1080/14787210.2021.1855143. 33249873 10.1080/14787210.2021.1855143

[16] Gumber D, Wang LD. Improving CAR-T immunotherapy: overcoming the challenges of T cell exhaustion. EBioMedicine. 2022;77:1–12. 10.1016/j.ebiom.2022.103941. 10.1016/j.ebiom.2022.103941 PMC8927848 35301179

[17] Zhang X, Zhang H, Lan H, Wu J, Xiao Y. CAR-T cell therapy in multiple myeloma: current limitations and potential strategies. Front. Immunol. 2023:1–13. 10.3389/fimmu.2023.1101495 10.3389/fimmu.2023.1101495 PMC9986336 36891310

[18] Sermer D, Brentjens R. CAR T-cell therapy: full speed ahead. Hematol Oncol. 2019;37(Suppl 1):95–100. 10.1002/hon.2591. 31187533 10.1002/hon.2591

[19] Dimitri A, Herbst F, Fraietta JA. Engineering the next-generation of CAR T-cells with CRISPR-Cas9 gene editing. Mol Can. 2022;21(1):1–13. 10.1186/s12943-022-01559-z. 10.1186/s12943-022-01559-z PMC8932053 35303871

[20] Srour SA, Akin S. Chimeric antigen receptor T-cell therapy for solid tumors: the past and the future. J Immunother Precis Oncol. 2023;6(1):19–30. 10.36401/JIPO-22-7. 36751657 10.36401/JIPO-22-7 PMC9888521

[21] Li W, Pan X, Chen L, Cui H, Mo S, Pan Y, Shen Y, Shi M, Wu J, Luo F, Liu J. Cell metabolism-based optimization strategy of CAR-T cell function in cancer therapy. Front Immunol. 2023;14(1186383):1–16. 10.3389/fimmu.2023.1186383. 10.3389/fimmu.2023.1186383 PMC10278966 37342333

[22] Pasvolsky O, Kebriaei P, Shah BD, Jabbour E, Jain N. Chimeric antigen receptor T therapy for adult B-cell acute lymphoblastic leukemia: state-of the-(C) ART and the road ahead. Blood Adv. 2023;7:3350–60. 10.1182/bloodadvances.2022009462. 36912764 10.1182/bloodadvances.2022009462 PMC10345854

[23] Benevolo Savelli C, Clerico M, Botto B, Secreto C, Cavallo F, Dellacasa C, Busca A, Bruno B, Freilone R, Cerrano M, Novo M. Chimeric antigen receptor-T cell therapy for lymphoma: new settings and future directions. Cancers. 2023;16(1):1–23. 10.3390/cancers16010046. 38201473 10.3390/cancers16010046 PMC10778255

[24] Wang C, Wang J, Che S, Zhao H. CAR-T cell therapy for hematological malignancies: History, status and promise. Heliyon. 2023;9(11):e21776. 10.1111/bjh.17544. 38027932 10.1016/j.heliyon.2023.e21776 PMC10658259

[25] Mitra A, Barua A, Huang L, Ganguly S, Feng Q, He B. From bench to bedside: the history and progress of CAR T cell therapy. Front Immunol. 2023;14:1188049. 37256141 10.3389/fimmu.2023.1188049 PMC10225594

[26] Mitra A, Barua A, Huang L, Ganguly S, Feng Q, He B. From bench to bedside: the history and progress of CAR T cell therapy. Front Immunol. 2023;15:14. 10.3389/fimmu.2023.1188049. 10.3389/fimmu.2023.1188049 PMC10225594 37256141

[27] Zhao Z, Chen Y, Francisco NM, Zhang Y, Wu M. The application of CAR-T cell therapy in hematological malignancies: advantages and challenges. Acta Pharm Sin B. 2018;8(4):539–51. 10.1016/j.apsb.2018.03.001. 30109179 10.1016/j.apsb.2018.03.001 PMC6090008

[28] Huang J, Huang X, Huang J. CAR-T cell therapy for hematological malignancies: limitations and optimization strategies. Front. Immunol. 2022:1–20. 10.3389/fimmu.2022.1019115. 10.3389/fimmu.2022.1019115 PMC9557333 36248810

[29] Sun W, Liang AB, Huang H, Huang XJ. Strategies to optimize chimeric antigen receptor T-cell therapy in hematologic malignancies: Chinese experience. Haematologica. 2023;108(8):2011–28. 10.3324/haematol.2022.282316. 36794504 10.3324/haematol.2022.282316 PMC10390786

[30] Shin S, Lee P, Han J, et al. Nanoparticle-based chimeric antigen receptor therapy for cancer immunotherapy. Tissue Eng Regen Med. 2023;20(3):371–87. 10.1007/s13770-022-00515-8. 36867402 10.1007/s13770-022-00515-8 PMC9983528

[31] Yang Z, Wang Y. Clinical development of chimeric antigen receptor-T cell therapy for hematological malignancies. Chin Med J (Engl). 2023;136(19):2285–96. 10.1097/CM9.0000000000002549. 37358555 10.1097/CM9.0000000000002549 PMC10538902

[32] Mahdi J, Dietrich J, Straathof K, et al. Tumor inflammation-associated neurotoxicity. Nat Med. 2023;29(4):803–10. 10.1038/s41591-023-02276-w. 37024595 10.1038/s41591-023-02276-w PMC10166099

[33] Li C, Xu J, Luo W, Liao D, Xie W, Wei Q, et al. Bispecific CS1-BCMA CAR-T cells are clinically active in relapsed or refractory multiple myeloma. Leukemia. 2024;2023(38):149–59. 10.1038/s41375-023-02065-x. 10.1038/s41375-023-02065-x PMC10776387 37848634

[34] Mailankody S, Devlin SM, Landa J, Nath K, Diamonte C, Carstens EJ, et al. GPRC5D-targeted CAR T cells for myeloma. N Engl J Med. 2022;387(13):1196–206. 10.1056/NEJMoa2209900. 36170501 10.1056/NEJMoa2209900 PMC10309537

[35] Ramezani F, Panahi Meymandi AR, Akbari B, et al. Outsmarting trogocytosis to boost CAR NK/T cell therapy. Mol Can. 2023;22(1):1–14. 10.1186/s12943-023-01894-9. 10.1186/s12943-023-01894-9 PMC10652537 37974170

[36] CorradoBenevolo Savelli, Clerico M, Botto B, Secreto C, Cavallo F, Chiara Dellacasa, et al. Chimeric antigen receptor-T cell therapy for lymphoma: new settings and future directions. Cancers. 2023;16(1):46–6.10.3390/cancers16010046 10.3390/cancers16010046 PMC10778255 38201473

[37] Amorós-Pérez B, Rivas-Pardo B, Gómez del Moral M, Subiza JL, Martínez-Naves E. State of the art in CAR-T cell therapy for solid tumors: is there a sweeter future? Cells. 2024 ;13(9):725. 10.3390/cells13090725 10.3390/cells13090725 PMC11083689 38727261

[38] De Marco RC, Monzo HJ, Ojala PM. CAR T cell therapy: a versatile living drug. Int J Mol Sci. 2023;24(7):6300. 10.3390/ijms24076300. 37047272 10.3390/ijms24076300 PMC10094630

[39] Sterner RC, Sterner RM. CAR-T cell therapy: current limitations and potential strategies. Blood Cancer J. 2021;11(4):1–11. 10.1038/s41408-021-00459-7. 33824268 10.1038/s41408-021-00459-7 PMC8024391

[40] Frey N, Porter D. Cytokine release syndrome with chimeric antigen receptor T cell therapy. Biol Blood Marrow Transplant. 2019;25(4):e123–7. 10.1016/j.bbmt.2018.12.756. 30586620 10.1016/j.bbmt.2018.12.756

[41] Zhang Y, Qin D, Shou AC, Liu Y, Wang Y, Zhou L. Exploring CAR-T cell therapy side effects: mechanisms and management strategies. J Clin Med. 2023;12(19):6124. 10.3390/jcm12196124. 37834768 10.3390/jcm12196124 PMC10573998

[42] Parikh RH, Lonial S. Chimeric antigen receptor T-cell therapy in multiple myeloma: a comprehensive review of current data and implications for clinical practice. CA Cancer J Clin. 2023;73(3):275–85. 10.3322/caac.21771. 36627265 10.3322/caac.21771

[43] Peters DT, Savoldo B, Grover NS. Building safety into CAR-T therapy. Hum VaccinImmunother. 2023;19(3):1–8. 10.1080/21645515.2023.2275457. 10.1080/21645515.2023.2275457 PMC10760383 37968136

[44] Chandran SS, Klebanoff CA. T cell receptor-based cancer immunotherapy: emerging efficacy and pathways of resistance. Immunol Rev. 2019;290(1):127–47. 10.1111/imr.12772. 31355495 10.1111/imr.12772 PMC7027847

[45] Sanber K, Savani B, Jain T. Graft-versus-host disease risk after chimeric antigen receptor T-cell therapy: the diametric opposition of T cells. Br J Haematol. 2021;195(5):660–8. 10.1111/bjh.17544. 34036558 10.1111/bjh.17544

[46] Savoldo B, Grover N, Dotti G. CAR T cells for hematological malignancies. The Journal of Clinical Investigation. 2024 Jan 16;134(2). 10.1172/JCI177160 10.1172/JCI177160 PMC10786683 38226627

[47] Ramos CA, Heslop HE, Brenner MK. CAR-T Cell Therapy for Lymphoma. Annu Rev Med. 2016;67:165–83. 10.1146/annurev-med-051914-021702. 26332003 10.1146/annurev-med-051914-021702 PMC4732525

[48] Czaplicka A, Lachota M, Pączek L, Zagożdżon R, Kaleta B. Chimeric antigen receptor T cell therapy for pancreatic cancer: a review of current evidence. Cells. 2024;13(1):1–16. 10.3390/cells13010101. 10.3390/cells13010101 PMC10777940 38201305

[49] Abbasi S, Totmaj MA, Abbasi M, et al. Chimeric antigen receptor T (CAR-T) cells: novel cell therapy for hematological malignancies. Cancer med. 2023;12(7):7844–58. 10.1002/cam4.5551. 36583504 10.1002/cam4.5551 PMC10134288

[50] Zmievskaya E, Valiullina A, Ganeeva I, Petukhov A, Rizvanov A, Bulatov E. Application of CAR-T cell therapy beyond oncology: autoimmune diseases and viral infections. Biomedicines. 2021;9(1):1–13. 10.3390/biomedicines9010059. 10.3390/biomedicines9010059 PMC7827151 33435454

[51] Charrot S, Hallam S. CAR-T Cells: future perspectives. Hemasphere. 2019;3(2):1–18. 10.1097/HS9.0000000000000188. 31723827 10.1097/HS9.0000000000000188 PMC6746028

